# Surmounting Chemotherapy and Radioresistance in Chondrosarcoma: Molecular Mechanisms and Therapeutic Targets

**DOI:** 10.1155/2011/381564

**Published:** 2010-12-30

**Authors:** Anne C. Onishi, Alexander M. Hincker, Francis Y. Lee

**Affiliations:** Department of Orthopaedics, College of Physicians and Surgeons, Columbia University, Black Building, 14-1412, 650 West 168th Street, New York, NY 10032, USA

## Abstract

Chondrosarcoma, a primary malignancy of bone, has eluded successful treatment with modern chemotherapeutic and radiation regimens. To date, surgical resection of these tumors remains the only curative treatment offered to patients with this diagnosis. Understanding and exploring the nature of chemotherapy and radiation resistance in chondrosarcoma could lead to new molecular targets and more directed therapy for these notoriously difficult-to-treat tumors. Here we review the most current hypotheses regarding the molecular mechanisms mediating chemotherapy and radiation resistance and the future direction of chondrosarcoma therapy research.

## 1. “Know thy Enemy”: Treatment Obstacles in Patients with Chondrosarcoma

Tzu, the 6th century BC Chinese war theorist, is most famous for his military treatise* The Art of War* and for having written, “Know thyself, know thy enemy; one thousand battles, one thousand victories” [1]. Chondrosarcoma, a heterogeneous group of tumors with extraordinarily diverse presentations and morphologies that have an enormous range of clinical behaviors, is a complex and difficult enemy to know. To date, patients who receive a diagnosis of chondrosarcoma are treated primarily with wide resective surgery since these tumors are notoriously resistant to both chemotherapy and radiation treatment. Dramatic excisions and even amputations are required, since local control of tumor is of paramount importance in order to prevent future metastasis. 

Chondrosarcoma is the second most common type of primary bone malignancy in the United States after osteosarcoma. It represents 26% of all primary bone cancers, with approximately 2000 new cases every year [2]. These tumors are characterized by cartilage-forming cells with no evidence of direct osteoid formation [3] and are graded based on local invasiveness and metastatic potential. They can be further classified by histologic characteristics such as cellularity, matrix changes, cell character, and replicative activity [4]. Clinically, the most common sites of tumor formation include the pelvis and appendicular long bones, though reports in the literature have documented other sites including the distal appendicular bones, temporomandibular region [5], and thoracic spine [6].

While wide resection with limb salvage surgery or amputation are definitive therapies for patients presenting with appendicular tumors [7], such surgeries introduce disability and morbidity into the lives of patients. Furthermore, sufficiently wide resection is not always possible in large tumors, tumors growing in the pelvis or axial skeleton, or tumors that have already metastasized. In such cases, tumor location, chemoresistance, and radioresistance lead to insurmountable treatment obstacles and very poor outcomes. Understanding the molecular mechanisms of resistance to chemotherapy and radiation in these tumors could lead to new targets for treatment or adjuvant therapies to surgery, thereby improving the length and quality of life in patients suffering from chondrosarcoma. 

In this paper, we will provide a summary of the most recent research regarding the molecular pathways and genes responsible for chemotherapy and radiation resistance in chondrosarcoma tumors (see Table 1). We will also explore the implications of these molecular mechanisms with respect to their role in the development of new therapies for this difficult-to-treat cancer. 

## 2. The Expression of P-Glycoprotein Allows Chondrosarcoma Cells to Withstand the Cytotoxic Effects of Most Chemotherapy Agents

P-glycoprotein is an ATP-dependent membrane-bound pump that excretes small hydrophobic molecules and is normally expressed in cells with secretory functions such as the proximal tubular cells of the kidney and the epithelial lining of the bile duct. It is also expressed in the hypertrophic region of the epiphyseal growth plate, providing additional evidence for its important physiologic excretory rolein protecting the cell from extracellular insults [8].

**Figure 1:**
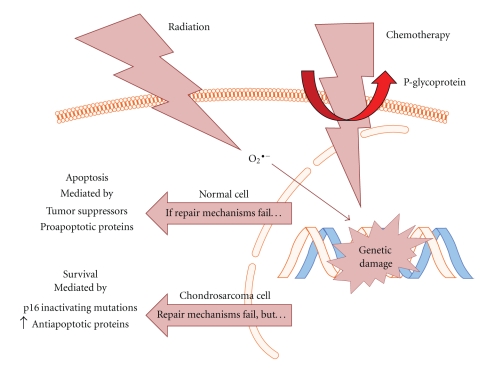
An overview of how chondrosarcoma cells evade the cytotoxic effects of chemotherapy and radiation. In normal cells, radiation and chemotherapy cause cell death by inducing genetic damage either directly, or in the case of radiation, through a reactive oxygen species (ROS) intermediate. For example, the drug doxorubicin intercalates with DNA, preventing replication, while ROS cause strand breaks. This damage is sensed by the cell, and then through the actions of tumor suppressor proteins such as p16 or p53 and the proapoptotic proteins including Bax, Bak, and Bim, the cell undergoes apoptosis, becomes senescent, or necroses. The chondrosarcoma cell's main defense against chemotherapeutic agents is P-glycoprotein, a membrane-bound pump that extrudes small, hydrophobic molecules from within the cell [10]. The action of P-glycoprotein can lower intracellular concentrations of chemotherapeutic agents beyond a point at which they exact their cytotoxic effects. Though radiation treatment still induces genetic damage in chondrosarcoma cells, several mutations allow them to survive. These mutations include inactivation of the gene encoding the important tumor suppressor p16 via methylation or deletion [11], and upregulation of the antiapoptotic proteins Bcl-2 and XIAP [12]. Figure adapted from Motifolio Cell and Nucleic Acid Toolkit.

The expression of P-glycoprotein in chondrosarcoma tumors has been well-established, and it has been proposed that this expression is an extremely important mechanism in the development of chemoresistance (see Figure 1). P-glycoprotein is encoded by the gene Multiple Drug Resistance-1, or *MDR-1*. Expression of this protein is common in both benign and malignant cartilaginous lesions, and in one study, 90% of tumors stained with specific antibodies were positive for P-glycoprotein expression [9]. The authors postulated that *MDR-1* expression was a marker for the presence of drug resistance to multiple chemotherapy agents including cyclophosphamide, doxorubicin, vincristine, cisplatin, methotrexate, and dacarbazine.

It has been shown using fluorescence microscopy and other *in vitro *techniques that chondrosarcomacells expressing P-glycoprotein accumulate lower levels of intracellular doxorubicin thanchondrosarcoma cells that do not express P-glycoprotein, and that the P-glycoprotein-positive cells are insensitive to the cytotoxic effects of doxorubicin at physiologic concentrations *in vitro* [10]. Rosier et al. have postulated that the development of chemoresistantchondrosarcoma tumors is a result of selective pressure against P-glycoprotein-negative cells in the presence of cytotoxic agents [8]. Alternatively, some authors suggest that toxin exposure causes direct upregulation of P-glycoprotein expression [13].

More recently, the importance of P-glycoprotein in mediating chemoresistance has been highlighted through experiments in which P-glycoprotein was inhibited both pharmacologically and with gene silencing techniques. Using siRNA, Kim et al. showed that knocking down the expression of *MDR-1* increased chemosensitivity by up to 4.1 fold [12]. Of note, a combination of the inhibition of P-glycoprotein with the inhibition of the antiapoptotic proteins Bcl-2, Bcl-xL, and XIAP showed an even higher increase in chemosensitivity, up to 5.5 fold [12]. These findings suggest that though P-glycoprotein is clearly an important mediator of chemoresistance in chondrosarcoma cells; other mechanisms are also involved.

## 3. Telomerase Activity Mediates Chondrosarcoma Cell Immortality

DNA polymerase cannot faithfully complete replication to the end of the superstructure of the macromolecule, which leads to the loss of nucleotides with each replication cycle—this is known as the “end replication problem.” Telomeres are regions of noncoding DNA at the end of the double strand that protect coding regions from this degradation. With every newgeneration, a piece of the telomere is lost, eventually causing the cell to exit the replication cycle [14]. Germ cells express telomerase, an enzyme capable of synthesizing new telomeres via its own RNA template, thereby allowing telomeres to be faithfully replicated and preventing the cell from exiting the replication cycle. Many cancer cell types including testicular, ovarian, breast, endometrial, and basal cell carcinoma and lymphoma express telomerase, and this is one suggested mechanism of attaining cellular immortality [15].

Many chondrosarcoma cells also express telomerase, and it has been established that telomerase expression in chondrosarcoma correlates significantly with tumor grade and rates of recurrence. Martin et al. have proposed that using immunohistochemical techniques to identify those tumors that express telomerase could be a useful adjuvant to traditional prognostic grading systems [16]. Dr. Martin's group has also explored the role of telomerase combined with the loss of tumor suppressor protein p16 as a means of malignant transformation of cartilage neoplasms and found that these two mutations in combination lead to a more aggressive and invasive phenotype *in vitro* [17].

Because of the important relationship between telomerase expression and cancer cell immortality, inhibition of telomerase has been an expanding area of cancer therapy research. It is hypothesized that by preventing the continued elongation of telomeres, malignant cells can reenter the normal cell cycleand and undergo the normal genetic and molecular checks on replication. Additionally, these cells would become susceptible to the apoptotic mechanisms that normally execute the process of programmed cell death when genetic damage is induced by chemotherapeutic agents.

The specific pharmacologic inhibitor of telomerase, BIBR1532, has been tested as a means to resensitize chondrosarcoma cells to traditional modes of chemotherapy. Parsch et al. have established telomerase expression in the chondrosarcoma cell line SW1353 and used the TRAP assay to show that BIBR1532 inhibits telomerase in SW1353 cells. Furthermore, it was demonstrated that long-term dosage of the telomerase inhibitor slowed the rate of growth of SW1353 cells, but did not arrest growth completely; the authors contend that this incomplete growth arrest is caused by chemotherapy-induced natural selection of clones with strong telomerase activity. Moreover, data from this study show that cell lines with low-telomerase activity were more sensitive to cisplatin-induced apoptosis while cell lines with high levels of telomerase activity were nearly resistant [18].

In clinical trials, telomerase inhibition by the specific inhibitor GRN 163L has been shown to have additive or even synergistic effects when used in combination with other chemotherapies and radiation regimens against leukemia [19]. These promising clinical results taken together with the aforementioned *in vitro* studies suggest that telomerase inhibition may be a beneficial strategy in the treatment of chemoresistant chondrosarcoma.

## 4. Targeting Angiogenesis in Chondrosarcoma Slows Tumor Growth

One important area of cancer research has been the role of angiogenesis in tumor growth and metastasis. As tumors grow beyond a few millimeters in size, oxygen can no longer diffuse to every cell, and the tumor becomes hypoxic. Angiogenesis, or the growth of new blood vessels, must occur for the tumor to grow beyond a certain size. Hypoxia induces angiogenesis via increased levels of the transcription factor hypoxia inducible factor-1*α* (HIF-1*α*). This small moleculeactivates the transcription of several proangiogenic factors, the most important of which is the cytokine vascular endothelial growth factor (VEGF). Because tumors depend on angiogenesis for survival and further growth, this pathway has become a popular drug target in many cancers, and chondrosarcoma is no exception.

Immunostaining and siRNA experiments have been used to demonstrate that chondrosarcoma tumors of all grades express HIF-1*α* at higher levels than do normal chondrocytes and benign cartilaginous tumors [20], and that VEGF expression is dependent on HIF-1*α* [21]. Another study concluded that in chondrosarcoma, HIF-1*α* expression was only present in 40% of tumors sampled; however, this study was unique in staining for nuclear HIF-1*α*, the active form. Tumors positive for nuclear HIF-1*α* were associated with decreased disease-free survival, suggesting that nuclear HIF-1*α* could serve as a prognostic factor in addition to histologic grade [20].

In terms of therapeutic potential, inhibiting angiogenesis may be a promising strategy for inducing growth arrest as an adjuvant to surgical removal of chondrosarcoma tumors. Klenke et al. have demonstrated that inhibition of the angiogenic tyrosine kinase receptors for VEGF, fibroblast growth factor (FGF), and platelet-derived growth factor (PDGF) with the small-molecule inhibitor SU6668 has a beneficial effect on tumor size and neovascular density *in vivo*. In this study, mice with chondrosarcoma xenografts implanted in the calvarium and treated with SU6668 had tumors that were 53% smaller than those tumors in mice treated with control injections at the end of the 28 day growth period. Additionally, tumors in mice treated with the inhibitor had vessel densities reduced by 37% compared to untreated mice [22]. In another *in vivo*   xenograft model, ecteinascidin-743 (ET-743), a sea squirt-derived chemotherapeutic, elicited profound tumor necrosis and prevented neovascularization when used in combination with plasminogen-related protein B, an endogenous molecule that downregulates endothelial cell metabolism [23].

## 5. Beyond Traditional Chemotherapy: Alternative Drug Targets in Chemoresistant Chondrosarcoma

Though they present unique treatment obstacles, chondrosarcoma tumors also present unique treatment opportunities because they maintain many of the phenotypic characteristics of the chondrocytes from which they are derived. One interesting area of research involves treating these phenotypic similarities as drug targets. Some exciting examples of these targets include cyclooxygenase-2 (COX-2) expression, melovonate synthesis, and estrogen signaling.

Chondrocytes are known to express the inflammatory cytokine COX-2 when exposed to other inflammatory cytokines and free radicals [24]. COX-2 expression has been demonstrated in peripheral chondrosarcoma tumors in several studies, and higher levels of expression are associated with poor prognosis [25, 26]. Schrage et al. hypothesized that there may be a role for COX-2 inhibition in chondrosarcoma treatment. Dr. Schrage demonstrated that the COX-2 inhibitor Celecoxib decreased cell viability *in vitro*. Celecoxib slowed tumor growth *in vivo* initially. Interestingly, these mechanisms were independent of COX-2 activity since several cell lines showed these responses but did not express COX-2 according to ELISA assays [25]. Further study is needed in this area because the *in vivo* growth arrest relapsed after six weeks of treatment.

Like Celecoxib, bisphosphonates are typically used in the treatment of other musculoskeletal conditions, but may have indications in the treatment of chondrosarcoma. Bisphosphonates inhibit melovonate synthesis and are extremely specific to the bone microenvironment. The mechanism of action involves inhibiting osteoclast-mediated bone resorption and the promotion of osteoblast differentiation. Interestingly, bisphosphonates may have an anticancer effect by inducing apoptosis, inhibiting metalloproteinase activity, and reducing VEGF levels [27]. It has been shown that the bisphosphonate zoledronic acid acts synergistically with paclitaxel in osteosarcomas [28]. In the context of chondrosarcoma, one case report demonstrates that zoledronic acid significantly reduces bone pain and improves the quality of life of patients with chondrosarcoma and chordoma [29]. Furthermore, another study showed that the third generation bisphosphonate minodronate decreases chondrosarcoma cell growth in a dose-dependent manner *in vitro* [30].

Finally, the role of estrogen signaling in skeletal development has implications for chondrosarcoma treatment. Estrogen signaling is involved in longitudinal bone growth, chondrocyte differentiation, and epiphyseal growth plate maturation. Therefore, Cleton-Jansen et al. hypothesized that targeting estrogen signaling could inhibit chondrosarcomacell proliferation. Dr. Cleton-Jansen demonstrated that inhibiting estrogen signaling with the aromatase inhibitor exemestane inhibits chondrosarcoma cell growth *in vitro* [31]. Estrogen signaling has been an important target in other cancers such as breast carcinoma, and this study suggests that it may also be an important target in chondrosarcoma.

## 6. Radiation Resistance in Chondrosarcoma

Radiation treatment is used widely and commonly in cancer therapy and incites its cytotoxic effects via the production of reactive oxygen species (ROS). These ROS then cause strand breaks in DNA. Under normal physiologic conditions, healthy cells have DNA repair machinery that senses ROS-induced genetic damage and initiates attempts to repair this damage (see Figure 1). If the damage is irreparable, proteins called tumor suppressors will signal to the cell to stop dividing and the cell will either undergo apoptosis, necrose, or become senescent. These mechanisms prevent genetic damage from being passed to future generations.

The efficacy of radiation treatment is contingent upon several things: first, formation of ROS is necessary to mediate genetic damage; second, the genetic damage-sensing mechanisms must be intact; third, the tumor suppression activity of the cell must be functional. Radiation resistance in tumor cells could feasibly develop at any one of these three steps due to mutations in the myriad proteins involved in these complex cascades. The exact mechanisms of radiation resistance in chondrosarcoma have remained somewhat unclear, though several culprits, which will be reviewed here, have been identified.

## 7. Loss of Tumor Suppressor p16 in Chondrosarcoma Leads to Radioresistance

The role of the loss of tumor suppressor genes is one well-known cause of radioresistance in numerous tumors. In chondrosarcoma, it has been shown that genetic changes to the p16 coding gene *CDKN2* are quite common in high-grade tumors. Asp et al. have shown using PCR and gene sequencing that 41% of chondrosarcoma tumors sampled had some sort of change to *CDKN2*, while no tumors sampled had any change to the well-known p53 tumor suppressor gene [11]. In addition, almost all of the highly malignant tumors sampled had alterations to this gene, suggesting that changes to the *CDKN2* gene are one important mechanism contributing to high malignancy. Such mutations to p16 are not seen in benign cartilaginous tumors such as enchondroma, suggesting a role for this gene in the transition to an aggressive, malignant phenotype [32]. These findings support evidence linking altered p16 expression to malignant phenotypes in other types of cancer including breast carcinoma and oropharyngeal squamous cell carcinoma [33, 34]. 

The role of p16 in mediating radioresistance was demonstrated by experiments that showed that reintroducing p16 expression with a viral vector can increase radiosensitivity *in vitro* by inciting mitotic catastrophe [35]. While no method of restoring the functionality of the p16 pathway has been developed *in vivo*, research is underway to use the defective p16/retinoblastoma (RB) pathway as the marker for targeting viral vectors to cancer cells. A virus could theoretically be derived that would only infect cells with a defect in the pathway; alternatively, a virus could be designed that would infect indiscriminately but would only replicate and/or produce its immune-modulatory product in tumor cells [36].

 Of course, treatments using viral vectors present their own issues such as ensuring viral genomic stability and selectivity for cancer cells versus healthy cells. Early trials of the use of such an oncolytic virus in the treatment of many types of cancer are currently underway. This particular adenovirus selectively targets cells with p16/RB pathway dysfunction and stimulates the production of granulocyte-macrophage colony-stimulating factor (GM-CSF), an immune modulator that locally induces CD8+ T cell and natural killer (NK) cell activity. Though the initial results suggest that the virus is capable of increasing tumor chemosensitivity without causing severe side effects, these trials have not included chondrosarcoma patients [37].

## 8. The Antiapoptotic Proteins Bcl-2, Bcl-xL, and XIAP Can Mediate Radioresistance

Within any cell, survival and proliferation are mediated by a delicate balance between pro- and antiapoptotic signals. When the balance is tipped in favor of antiapoptotic signals, mediated by proteins such as Bcl-2, Bcl-xL, and XIAP, the cell will survive, proliferate, differentiate, and migrate. Conversely, when the balance is tipped in favor of proapoptotic signals, such as Bax, Bak, and Bim, the cell will exit the cell cycle, disrupt its nucleus, and begin to break apart into small parts commonly referred to as “blebs.” 

Given that ionizing radiation causes cytotoxicity by inducing apoptosis, one theory regarding radiation resistance is that some cancer cells may tip their own internal balance to favor the anti-apoptosis signals. That way, no matter how much genetic damage is induced by radiation, the cell will continue to differentiate, divide, and survive. 

Kim et al. have shown that chondrosarcoma cells express higher basal levels of the antiapoptotic proteins Bcl-2 and XIAP than do normal chondrocytes *in vitro*, though the two cell types express similar amounts of the antiapoptotic protein Bcl-xL [38]. In addition, radiation induces increased expression of these proteins in a dose-dependent manner, suggesting an important role of antiapoptotic signaling in radioresistance [38]. Finally, Dr. Kim demonstrated an increase in radiosensitivity by using siRNA to silence the expression of these proteins—silencing them individually could lead to up to 9.2 fold increases in radiation sensitivity. Silencing two of these genes simultaneously enhanced sensitivity to 5 and 10 Gy doses of radiation by 11.3 and 11.2 fold, respectively [38].

Clinically, gene silencing of Bcl-2 has great potential as an adjuvant therapy. In Phase III trials, it has already been shown that the antisense oligonucleotide G3139 combined with the chemotherapy agent dacarbazine increased progression-free and overall survival in patients with advanced melanoma when compared to patients treated with just dacarbazine alone [39]. Such clinical studies have shown that treating humans with antisense oligonucleotides against Bcl-2 is feasible and potentially beneficial.

Little is known about the possible additive or synergistic effects of Bcl-2 antisense oligonucleotides in the context of radiation treatment, as no clinical trials have been undertaken. Several studies have shown promising results in xenograft models: Bcl-2 antisense oligonucleotides have inherent antitumor activity and can enhance the effects of local tumor irradiation in a colon carcinoma model [40] and in a nasopharyngeal carcinoma model [41].

Given these findings, it would appear that targeting the expression of these antiapoptotic proteins is a promising new avenue of inducing radiation sensitivity in chondrosarcoma tumors. Previous clinical studies have already established the feasibility and safety of antisense oligonucleotide treatment and pharmacologic inhibition in humans, though *in vivo* studies in a chondrosarcoma model are lacking.

## 9. Technological Advances in Radiation Therapy: Improving Outcomes in Chondrosarcoma

Many improvements in the field of radiation oncology in general can be applied to treatment of chondrosarcoma. In short, these advances can be classified into those that allow the delivery of greater amounts of radiation to the tumor and those that enhance the killing power of a given amount of radiation. With regard to the former category, improvements in proton therapy have allowed the safe delivery of more radiation to tumors. Unlike traditional radiotherapy, proton therapy does not deliver radiation to tissue beyond the tumor [42]. This is especially important in tumors occurring in areas such as the spine and the skull (tumors which are traditionally very difficult to resect with a wide margin and, as such, most often in need of radiation). Additional advances in this technology have allowed increased focusing of the radiation (“spot-scanning” technology), allowing the use of more intense radiation without increasing the toxicity to the patients. This technology was recently tested in chondrosarcomaof the skull base, and the results suggest that the technique is relatively safe and can be an effective adjuvant to surgery when used at high doses [43].

In addition to delivering more radiation directly to tumors, improving the efficiency of a given dose of radiation is another strategy for dealing with radioresistant tumors. Because of the hypoxic conditions inherent to the microenvironment of a chondrosarcoma tumor, the generation of ROS is severely curtailed. Acridine Orange (AO) is capable of producing damaging oxidants in the presence of gamma radiation even in oxygen-poor environments. This capability makes AO an attractive addition to radiotherapy for chondrosarcoma. Moussavi-Harami et al. have shown that AO in and of itself is only slightly cytotoxic but induces significant increases in sensitivity to low-dose radiation *in vitro *[35].

## 10. Conclusion

We have reviewed here the numerous mechanisms that make chondrosarcoma a challenging cancer to treat. As Tzu suggested, knowing the enemy is a vital part of any successful confrontation. Clinical experience has shown us that chondrosarcoma is strongly resistant to traditional means of cancer treatment, save for dramatic surgeries. Yet Tzu also notes that “the way to avoid what is strong is to strike at what is weak” [1]. Cutting edge research in the field of chondrosarcoma has exposed some of these key weaknesses. Exploiting these vulnerabilities will allow us to undermine the resistance that has made these tumors such a frustrating clinical entity and to offer much needed treatment options to patients who would otherwise face a very poor prognosis.

## Figures and Tables

**Table 1:** A summary of the mechanisms involved in chemotherapy and radiation resistance in chondrosarcoma cells. Summarized here are the various molecular mechanisms responsible chemotherapy and radiation resistance in chondrosarcoma, strategies to overcome these mechanisms, and the results of studies testing these strategies as possible adjuvants to traditional surgical treatments. See text for more in-depth discussions of each topic.

Resistance mechanism	Effect within the cell	Therapeutic strategies	Has treatment been tested?
P-glycoprotein expression	Exports chemotherapy drugs from within the cell	Gene silencing using siRNA pharmacologic inhibition using C-4	*In vitro*: dramatic increases in chemosensitivity

Telomerase activity	“Immortal” cell phenotype	BIBR1532- pharmacological telomerase inhibitor	BIBR152- *in vitro*: slowed growth and increased sensitivity to cisplatin.
		GRN 163L- pharmacological telomerase inhibitor	GRN 163L- *in vivo*: but not in chondrosarcoma—helpful addition in leukemia treatment

Angiogenesis	Supports larger tumors, allows metastasis	SU6668-inhibits receptors for VEGF, FGF, PDGF ET-743 and plasminogen-related protein B-chemotherapeutic and endothelial-cell-metabolism downregulator	*In vivo* murine model: decreased tumor size, vascularization.
			*In vivo* murine model:induction of profound necrosis, inhibition of neovascularization

COX-2 expression	Unclear, but associated with poor prognosis	COX-2 inhibitors-mechanism of action remains unclear	*In vivo: *slows cancer growth, although growth relapses after six weeks of treatment

Melovonate synthesis	Shift bone remodeling balance toward resorption	Bisphosphonates-inhibit melovonate synthesis in the bone microenvironment	*In vivo*: induction of apoptosis, inhibition of metalloproteinase activity, and reduction of VEGF levels

Tumor Suppressor p16 Mutation	Decreased tendency toward apoptosis	p16-restoring virusOncolytic viruses-selectively target immune-inducing molecules to cells with pathway defects	*In vitro*: increased radiosensitivity in p16-deficient cells.
			*In vivo*, but not in chondrosarcoma-results show strong selectivity, desired efficacy, no serious side effects.

Increased expression of Bcl-2, Bcl-xL, and XIAP	Decreased tendency toward apoptosis	Downregulation via pharmacotherapy/siRNA-shift cellular balance toward apoptosis	*In vitro*: increased radiosensitivity
			*In vivo*: increased chemosensitivity

Hypoxia	Decreased ROS creation by radiation	Acridine orange-enhances ROS creation	*In vivo*: significantly increased radiosensitivity
